# The Anatomy of the Central Nervous System of Man and of Vertebrates in General

**Published:** 1899-06

**Authors:** 


The Anatomy of the Central Nervous System of Man and of Vertebrates in General. By Prof. Ludwig Edinger, M. D., Frankfort-on-the-Main. Translated from the Fifth German Edition by Winfield S. Hall, Ph. D., M. D., Professor of Physiology in the Northwestern Medical School, Chicago. Assisted by Philo Leon Holland, M. D., Instructor in Clinical Neurology in the Northwestern University Medical School, and Edward P. Carleton, B. S., Demonstrator of Histologic Neurology in the Northwestern University Medical School, Chicago. Illustrated, pp. XI.–446. Philadelphia: The F. A. Davis Co., 1914–16 Cherry street

A new translation from the German fifth edition of Edinger shows the popularity which this work has achieved. It is not often that a work on a special subject, not a necessity to the student or general practitioner, runs through five successive editions, and to do so must possess considerable merit. The book has grown with each edition until it is now fast approaching Schwalbe's classical work on the nervous system. The illustrations—as important as the text—are numerous enough, but are horribly executed and more of a detraction than attraction to the book. It is too bad that such illustrations as embellish Obersteiner, or Schwalbe, or even Féré, or those appearing in some of our American text-books of the nervous system cannot be substituted for Edinger's. We trust that in the future editions the same progress and improvement will extend to the plates as has extended to the text. The translator has done his work well and is to be congratulated. The book is well printed and nicely bound.

